# Bioactive glycoalkaloides isolated from *Solanum melongena* fruit peels with potential anticancer properties against hepatocellular carcinoma cells

**DOI:** 10.1038/s41598-018-36089-6

**Published:** 2019-02-11

**Authors:** Mostafa I. Fekry, Shahira M. Ezzat, Maha M. Salama, Ohoud Y. Alshehri, Ahmed M. Al-Abd

**Affiliations:** 1 Pharmacognosy Department, Faculty of Pharmacy, Cairo University, Cairo, Egypt; 2 Biomedical Research Section, Nawah Scientific, Mokattam, Cairo Egypt; 3 Department of Pharmacognosy, Faculty of Pharmacy, October University for Modern Science and Arts (MSA), 6th October, Giza, 12566 Egypt; 4 Pharmacognosy Department, Faculty of Pharmacy, The British University of Egypt, El-Sherouk city, Cairo, 11837 Cairo Egypt; 5 Research Centre, King Faisal Specialist Hospital and Research Center, Jeddah, 21499 Saudi Arabia; 6 Pharmacology Department, Medical Division, National Research Centre, Dokki, Giza, Egypt; 7 Department of Pharmaceutical Sciences, College of Pharmacy, Gulf Medical University, Ajman, 4184 Ajman United Arab Emirates

## Abstract

Hepatocellular carcinoma (HCC) is progressively increasing tumor with lack of accurate prognosis and inadequate systemic treatment approaches. Solanum sp. (such as *Solanum melongena*) is a folk herb which is reported to possess anticancer properties. In a continuity for our interest in pursuing the anticancer activity of compounds isolated from the fruit peels of *Solanum melongena*, the HPLC profiling and ESI-MS assessment for the methanolic extract evidenced the presence of bioactive glycoalkaloids (solasonine, solasodine and solamargine). These glycoalkaloids were isolated, purified and proved to possess *in vitro* cytotoxicity against human liver cancer cell lines (Huh7 and HepG2). Herein, we investigated the potential mechanism of action of these compounds using DNA content flow-cytometry and apoptosis/necrosis differential anaylsis using annexin-V/FITC staining. Solasonine, solasodine and solamargine inducd significant antiproliferative effect against liver cancer cells (Huh7 and HepG2) which was attributed to cell cycle arrest at S-phase. Solamargine, solasodine and solasonine induced significant apoptosis in Huh7 cells. Only solamargine-induced cell cycle arrest, was reflected as apoptotic cell killing effect against HepG2 cells. In conclusion, glycoalkaloids derived from *Solanum melongena* and particularly, solamargine are promising antiproliferative agents with potential anticancer effects.

## Introduction

Hepatocellular carcinoma (HCC) is progressively increasing solid tumor type with poor prognosis and inadequate systemic treatment approaches. In human, almost 80% of patients die within one year of HCC diagnosis. In men, it is considered the fifth most common cancer and the third leading cause of cancer related mortality^1,2^. Chronic inflammatory liver disease due to high-fat diet, alcohol consumption, and chronic infection such as hepatitis virus B and C are the most common leading causes of HCC. Hepatitis C virus infection is considered the principal risk factor for HCC in Egypt^3^. HCC comprises national health problem; its incidence rate in Egypt alone is significantly larger than those observed in both USA and the rest of Middle Eastern countries^4^. Tumor development is correlated to both an increase in cell proliferations and a decrease in programmed cell death. It is now clear that development and progression of various liver diseases are accompanied with minimal increase or decrease in hepatocyte apoptosis. This in turn leads to extending hepatocyte cell viability and accumulated genetic mutations^5^.

Compounds derived from natural origin such as, herbal products and other folk remedies draw great attention as a treatment modality for several illnesses such as, malegnancies^6^. Several natural compounds and phytochemicals represent milestone chemotherapeutic agents which showed significant anticancer effects such as paclitaxel, doxorubicin, vincristine and others^7^. Solanum sp. is a folk herb which is abundant in open fields. It is frequently reported for the treatment of several cancers such as, cervical carcinoma, breast cancer, melanoma and most interestingly, liver cancer^8–12^. In terms of folk use, Solanum sp. was used for the treatment of edema, mastitis, inflammatory disorders^13^ and fever besides its robust anti oxidant and cytoprotective effects^14,15^.

Glycoalkaloids are class of steroidal glycosides which are structurally diverse and display broad spectrum of biological activities such as antibacterial, anti-inflammatory, and anticancer activities^16^. It was found that both non-sugar and sugar moieties are essential for the glycoalkaloids biological activity^17^. The conjugates of solasodine aglycone showed anticancer activity against human colon and liver cancer cells^18^. In our previous study, we isolated five steroidal glycosides from the methanolic extract of *Solanum melongena* fruit peels (MEP). MEP along with the isolated compounds were tested *in vitro* against five human cancer cell lines; colon cancer cell line HCT116, larynx cancer cell line HEP2, breast cancer cell line MCF7, cervix cancer cell line HeLa and liver cancer cell line HepG2. Solasonine, solasodine, and solamargine demonstrated the most potent activity among the tested compounds. Remarkably, human liver cancer cell line (HepG2) was considerably sensitive to the aforementioned compounds^9^. Herein, we describe the HPLC profile of MEP to confirm the presence of those glycoalkaloids (solasonine, solasodine and solamargine) in MEP. The identity of the compounds was further confirmed by ESI-MS. Furthermore, we investigated in some details the antiproliferative/cytotoxic, cell cycle interfering and apoptosis inducing profile of the three biologically active glycoalkaloids (solasonine, solasodine and solamargine) against two liver cancer cell lines HepG2 and Huh-7 cells.

## Results

### HPLC-PDA Detection of solasodine, solasonine, and solamargine in MEP

**Figure 1:**
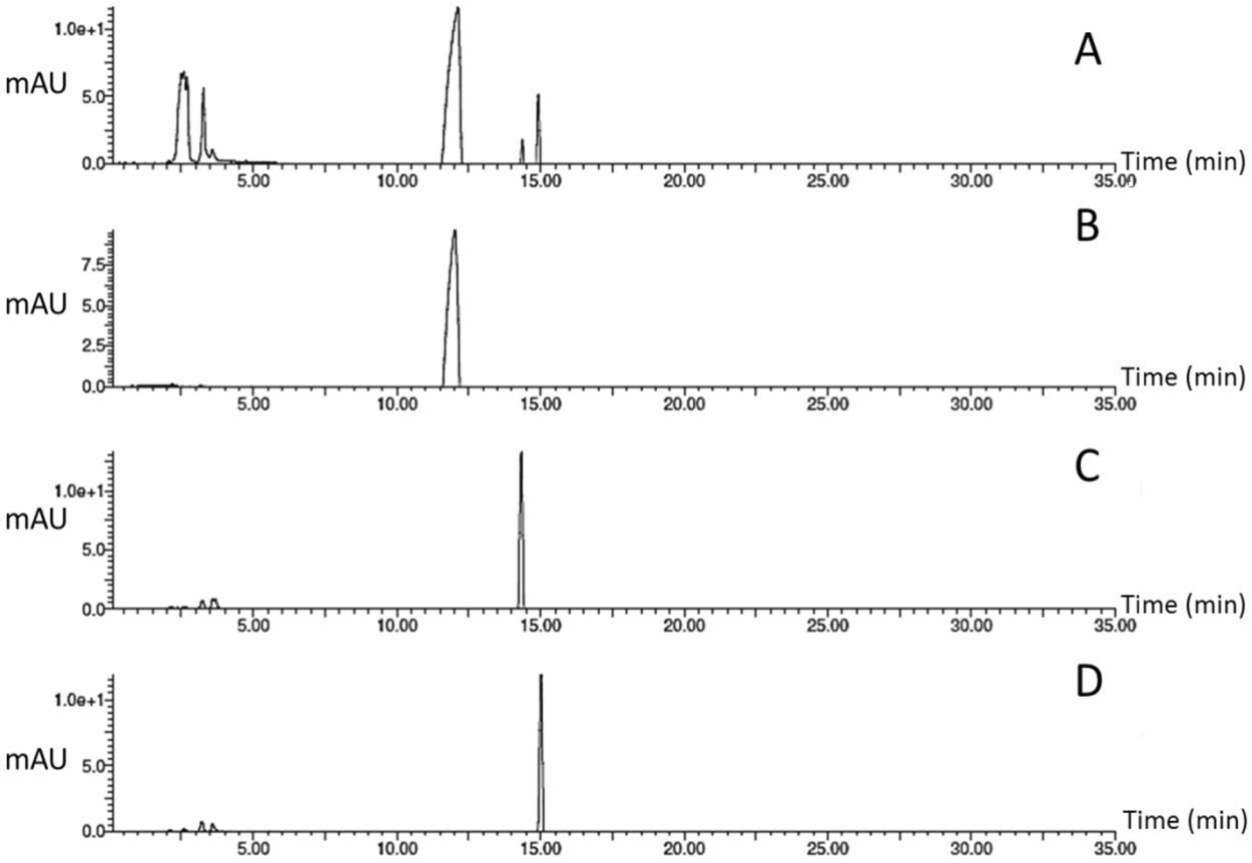
HPLC-PDA chromatogram of the methanolic extract of *Solanum melongena* fruit peels (**A**), solasodine standard (**B**) solasonine standard (**C**) and solamargine standard (**D**) monitored at 200 nm.

**Figure 2:**
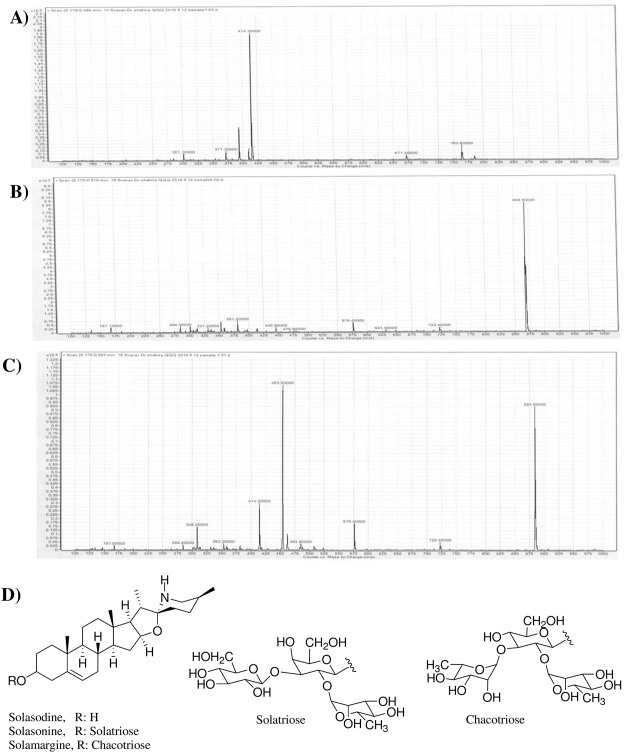
ESI-MS of solasodine (**A**), solamargine (**B**) and solasonine (**C**) in the positive ion mode. Structures of the isolated compounds from the MEP (**D**).

HPLC chromatograms *S. melongena* fruit peels (MEP) was monitored at 200 nm (Fig. 1-A). Solasodine, solasonine, and solamargine authentics have been injected using the same HPLC conditions to assign the chemical identity of the eluted peaks. Solasodine was first to be eluted from the MEP at 12 min (Fig. 1-B) followed by solasonine at 14.3 min (Fig. 1-C), then solamargine at 15 min (Fig. 1-D). The ESI-MS of the isolated compounds is displayed in Fig. 2(A–C). The structures of the isolated/tested compounds are displayed in Fig. 2-D.

### Dose response relationship of solasonine, solasodine and solamargine against liver cancer cells (huh-7 and HepG2

SRB-U assay was used to assess the cytotoxicity of the isolated glycoalkaloids against two different liver cancer cell lines (Huh-7 and HepG2) over concentration range of 0.01–100 μM. Tested compounds showed comparable cytotoxicity profile against both liver cell lines under investigation. However, Huh-7 cells were relatively more susceptible to cell killing effect copared to HepG2 cells.

**Figure 3:**
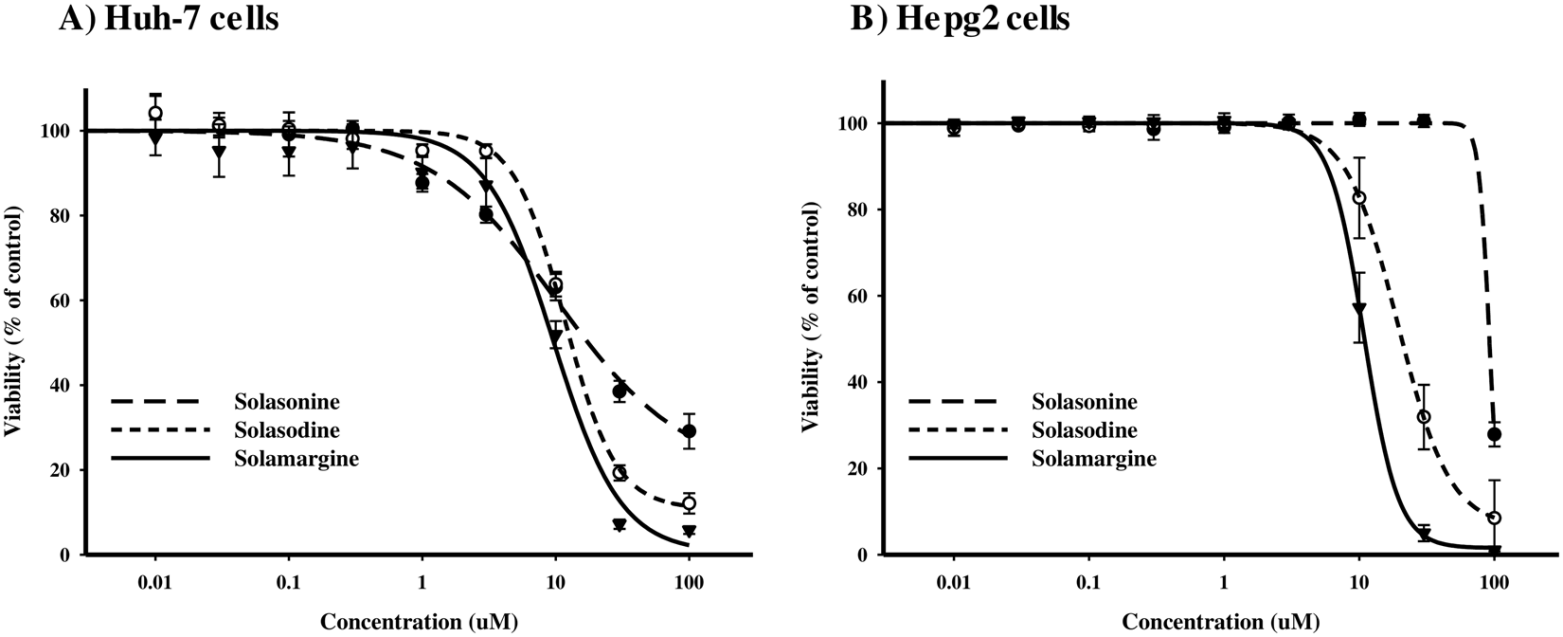
Dose response relationship of solasonine, solasodine and solamargine against Huh-7 (**A**) and HepG_2_ cells (**B**). Cells were exposed to serial concentrations of solasonine (●), solasodine (○) or solamargine (▼) for 72 h and viability was assessed by using SRB assay. Data are presented as mean ± SD (n = 3).

In Huh-7 cells, solasonine, solasodine and solamargine showed comparable cytotoxicity with IC_50_’s of 10.3 ± 1.5 μM, 11.7 ± 0.3 μM and 9.6 ± 0.5 μM, respectively. In addition, R-values (resistance fraction) for solasonine and solasodine were 19.2 ± 2.6% and 10.8 ± 1.0%, respectively. It is worth mentioning that, the R-value for solamargine was interestingly low (less than 1%) within Huh-7 cells (Fig. 3-A).

With respect to HepG2 cell line, solasonine showed significantly weaker cytotoxicity compared to Huh-7 cells with IC_50_ of 91.8 ± 9.4 μM. solasodine showed moderate cytotoxicity with IC_50_ equals 19.4 ± 0.4 μM. Interestingly, solamargine showed comparable potency against HepG2 cells with IC_50_ of 10.8 ± 0.1 μM. Similar to Huh-7 cells, solamargine did not suffer from any significant resistance (R-value was less than 1%) and none of the other two glycoalkaloids showed resistance value higher than 10% (Fig. 3-B.

### Influence of solasonine, solasodine and solamargine on the cell cycle distribution of Huh-7 cells

**Figure 4:**
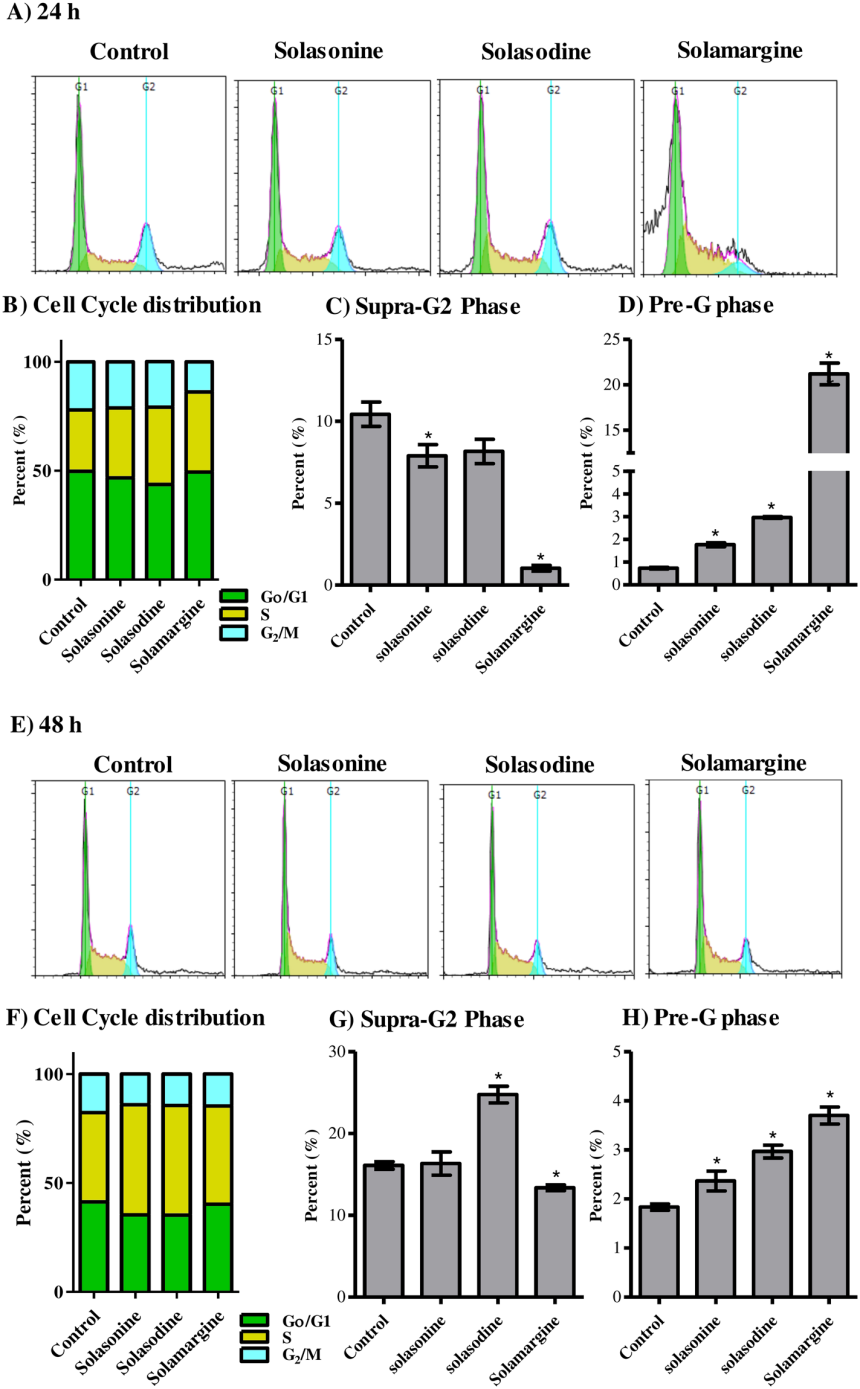
Effect of solasonine, solasodine and solamargine on the cell cycle distribution of Huh-7 cells. Cells were exposed to test compounds (10 µM) for 24 h (**A–D**) or 48 h (**E–H**)and cell cycle histograms were compared to control cells (**A**,**E**); and different cell cycle phases were plotted as percent of total events (**B**,**F**). Percentages of cells in pre-G_1_ phase (**C**,**G**) and supra-G_2_ phase (**D**,**H**) were assessed numerically and compared to control treatment. Data are presented as mean ± SD (n = 3); and (*) indicates significantly different from control group at P < 0.05.

In our previous work, solasonine, solasodine and solamargine showed promising anti-proliferative/cytotoxic effects against different cancer cell lines at concentration range of 1–10 µM. Herein, we further investigated the interference of these glycoalkaloids to cell cycle phases of Huh-7 liver cancer cells. Solasonoine and solasodine induced moderate cell cycle arrest at S-phase and increased percentage of cells in S-phase from 28.2 ± 1.6% to 32.1 ± 0.7% and 35.4 ± 1.8%, respectively. Despite cells accumulating in S-phase due to treatment with solasonine and solasodine, no significant change was noticed in G_2_/M-phase after treatment with both glycoalkaloid. On the other hand, S-phase arrest induced in Huh-7 cells due to solamargine (36.8 ± 3.1%) decreased cell propagation to G_2_/M-phase from 22.1 ± 3.0% to 13.9 ± 5.2% (Fig. 4-A,B). In alignment with the induced S-phase arrest, solamargine and, to a lesser extent, solasonine significantly decreased cell population in SupraG_2_-phase from 10.4 ± 1.3% to 1.0 ± 0.3% and 7.9 ± 1.2%, respectively (Fig. 4-C). In addition, solasonine, solasodine and solamargine significantly increased dead cell population (Pre-G phase) from 0.7 ± 0.1% to 1.8 ± 0.1%, 3.0 ± 0.1% and 21.1 ± 2.0%, respectively (Fig. 4-D).

Further exposure of Huh-7 cells to solasonine, solasodine and solamargine for 48 h resulted in further significant cell cycle arrest at S-phase; solamargine significantly increased S-phase cell population from 34.4 ± 0.9% to 43.9 ± 0.8% with reciprocal decrease in G_0_/G_1_-phase from 44.0 ± 1.3% to 31.5 ± 1.8%. In addition, solasonine and solasodine increased S-phase cell population to 39.6 ± 2.7% and 36.6 ± 0.4%, respectively. Only solamargine induced significant cell cycle arrest at G_2_/M-phase as well and increased cell population in G_2_/M-phase from 21.7 ± 0.5% to 24.6 ± 1.9% (Fig. 4-E,F). In addition and due to the induced G_2_/M-phase arrest, solamargine significantly decreased cell population in SupraG_2_-phase from 11.3 ± 1.2% to 6.8 ± 0. 8%. On contrary, solasonine increased cells in supraG_2_ phase to 15.0 ± 0.9% (Fig. 4-G). Moreover, solasodine and solamargine significantly increased dead cell population (Pre-G phase) from 1.3 ± 0.1% to 1.9 ± 0.1% and 8.5 ± 0.1%, respectively (Fig. 4-H).

To confirm our observations about cell cycle interference, more resistant liver cancer cell line (HepG_2_) was used.

### Influence of solasonine, solasodine and solamargine on the cell cycle distribution of HepG2 cells

**Figure 5:**
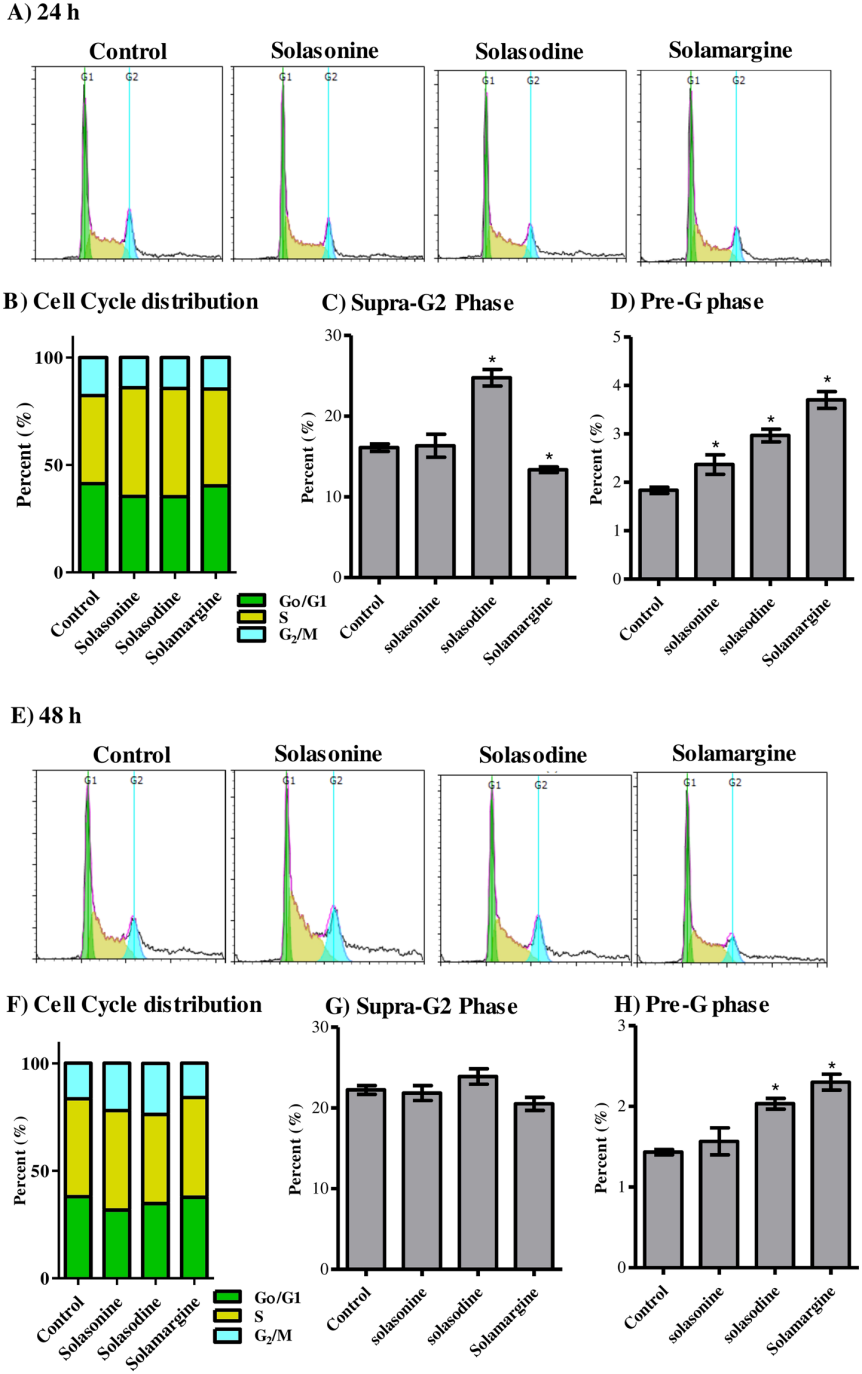
Effect of solasonine, solasodine and solamargine on the cell cycle distribution of HepG_2_ cells. Cells were exposed to test compounds ( 10 µM) for 24 h (**A–D**) or 48 h (**E–H**) and cell cycle histograms were compared to control cells (**A**,**E**); and different cell cycle phases were plotted as percent of total events (**B**,**F**). Percentages of cells in pre-G_1_ phase (**C**,**G**) and supra-G_2_ phase (**D**,**H**) were assessed numerically and compared to control treatment. Data are presented as mean ± SD (n = 3); and (*)indicates significantly different from control group at P < 0.05.

Besides Huh-7 cells, we further investigated the interference of these glycoalkaloids to cell cycle phases of the more resistant HepG2 liver cancer cells. Similar to Huh-7 cells, solasonine, solasodine and solamargine induced significant S-phase cell cycle arrest and increased percentage of cells in S-phase from 41.0 ± 0.3% to 50.6 ± 3.2%, 50.3 ± 1.0% and 45.0 ± 0.8%, respectively. Reciprocally, treatment with solasonine, solasodine and solamargine induced significant increase in cells in G_2_/M-phase from 17.7 ± 0.4% to 14.1 ± 1.3%, 14.4 ± 0.8% and 14.8 ± 0.3%, respectively; and significant decrease in G_0_/G_1_-phase from 41.4 ± 0.6% to 35.3 ± 2.0%, 35.2 ± 0.2% and 40.3 ± 0.6%, respectively (Fig. 5-A,B). Solasodine significantly increased cell population in SupraG_2_-phase from 16.1 ± 0.8% to 24.8 ± 1.7%; while solamargine decreased SupraG_2_-phase cells to 13.4 ± 0.6% (Fig. 5-C). Furthermore, solasonine, solasodine and solamargine significantly increased dead cell population (Pre-G phase) from 1.8 ± 0.1% to 2.3 ± 0.4%, 3.0 ± 0.2% and 3.7 ± 0.3%, respectively (Fig. 5-D).

Extended exposure of HepG_2_ cells to solasonine for 48 h resulted in significant cell cycle accumulation at G_2_/M-phase from 16.4 ± 0.9% to 22.0 ± 2.5%. Solasodine significantly increased G_2_/M-phase cell population from 16.4 ± 0.9% to 23.7 ± 1.7% with reciprocal decrease in G_0_/G_1_-phase from 38.0 ± 1.3% to 34.8 ± 1.1%; and in S-phase from 45.5 ± 1.4% to 41.5 ± 0.6%. (Fig. 5-E,F). The three tested glycoalkaloids showed non-significant change in supraG_2_ phase cells (Fig. 5-G). In addition, solasodine and solamargine significantly increased dead cell population (Pre-G phase) from 1.5 ± 0.1% to 2.0 ± 0.1% and 2.3 ± 0.1%, respectively (Fig. 5-H).

Further analysis for the mechanism of cell damage (programmed vs. non-programmed) induced by our test compounds were studied using annexin V-FITC/PI differential staining.

### Apoptosis/necrosis assessment using flow cytometry

**Figure 6:**
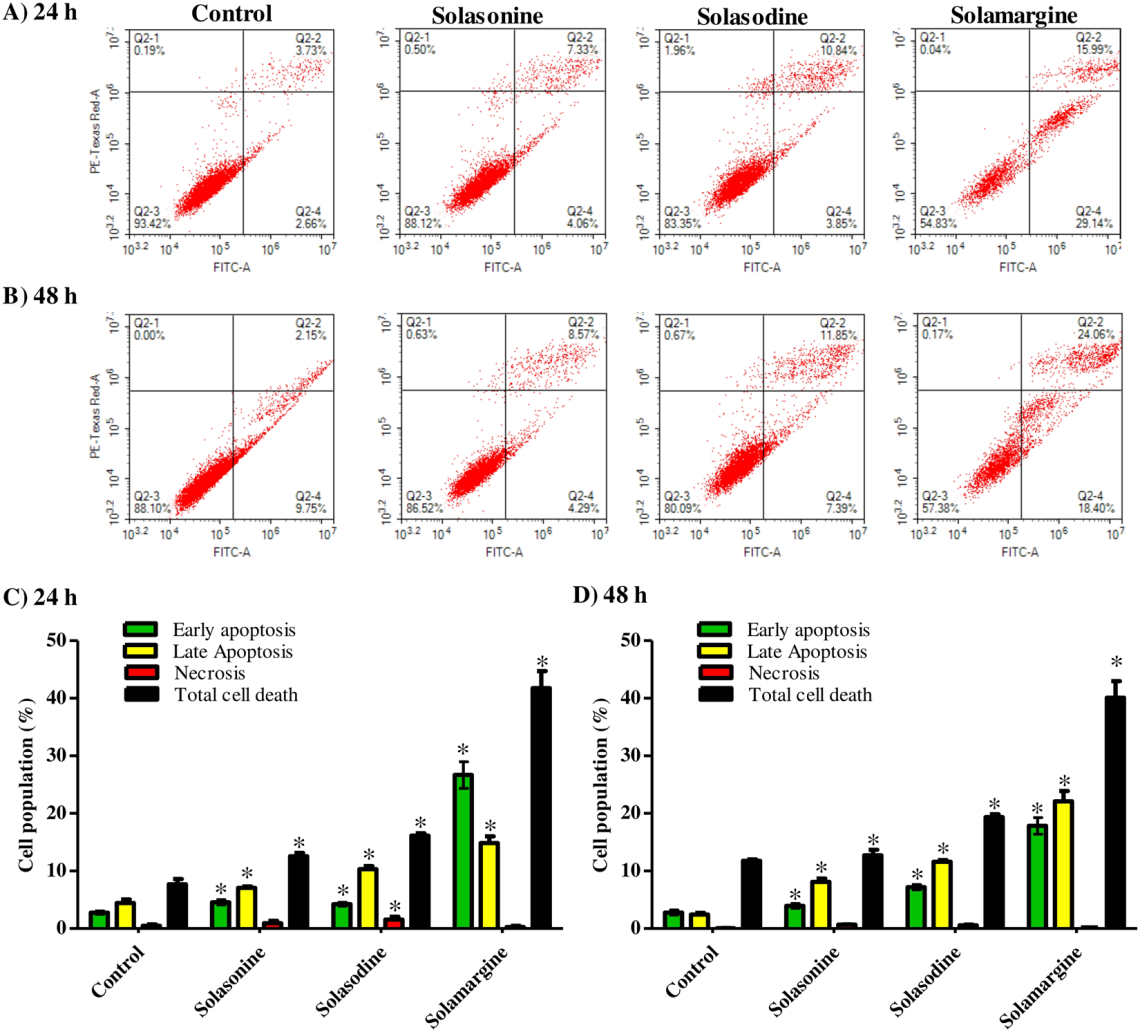
Cell death mechanism in Huh-7 cells after treatment with solasonine, solasodine and solamargine. Cells were exposed to 10 µM of solasonine, solasodine and solamargine for 24 (**A**) and 48 h (**B**) and percentages of cells undergoing apoptosis/necrosis were assessed using Annexin-V/FITC-PI staining and compared to control cells (**C**,**D**). Data are presented as mean ± SD (n = 3); and (*) indicates significantly different from control group at P < 0.05.

Annexin V-FITC/PI staining coupled with flow cytometry was used to differentially assess proportion of cells dying via necrosis versus cells undergoing apoptosis in both Huh-7 and HepG2 cells. Cells were treated with 10 μM of solasonine, solasodine and solamargine for 24 h and 48 h prior to apoptosis/necrosis differential assessment. In Huh-7 cells, all glycoalkaloids under investigation (solasonine, solasodine and solamargine) significantly increased apoptosis (early and late) by 1.6, 2 and 5.8 folds, respectively compared to control cells; and consequently increased total cell death. Only solasodine induced non-specific necrotic cell death (3.3 folds) in Huh-7 cells (Fig. 6-A,C). Further exposure of huh-7 cells to solasonine, solasodine and solamargine resulted in similar profile of apoptotic cell death; they significantl increased the apoptosis cell fractions (early and late) by 2.3, 3.6 and 7.6 folds, respectively compared to control (Fig. 6-B,D).

**Figure 7:**
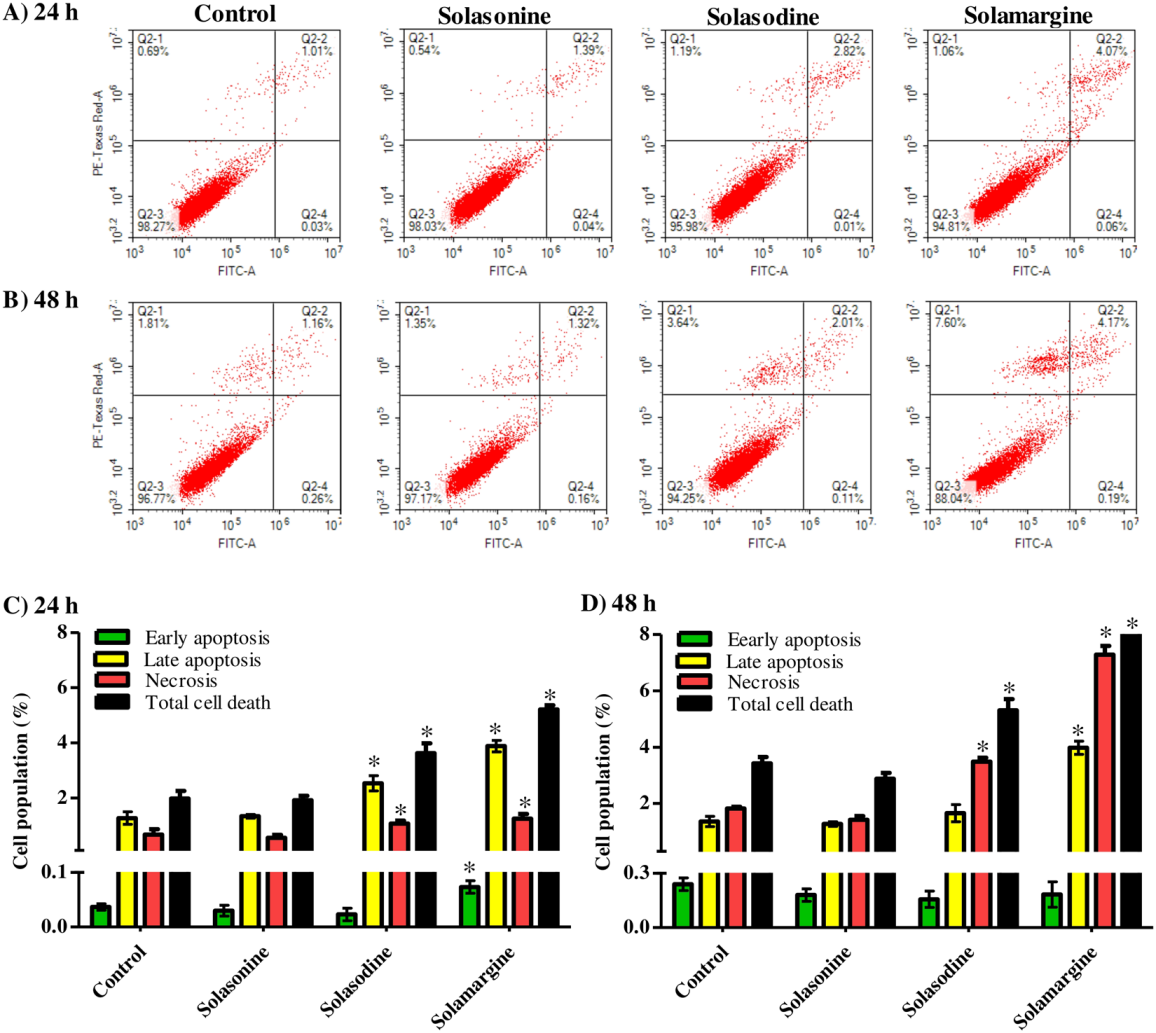
Cell death mechanism in HepG_2_ cells after treatment with solasonine, solasodine and solamargine. Cells were exposed to 10 µM of solasonine, solasodine and solamargine for 24 (**A**) and 48 h (**B**) and percentages of cells undergoing apoptosis/necrosis were assessed using Annexin-V/FITC-PI staining and compared to control cells (**C**,**D**). Data are presented as mean ± SD (n = 3); and (*) indicates significantly different from control group at P < 0.05.

With respect to HepG_2_ cells, only solamargine showed significantly more cells with early apoptosis compared to control untreated cells. In addition,solasodine and solamargine showed significant apoptosis induction (2 and 3 folds more late apoptosis cells compared to control untreated cells, respectively); and marginal overall killing effect (1.6 and 2.8 folds, respectively) after 24 h of exposure. (Fig. 7-A,C). Further exposure of HepG_2_ cells to solasodine and solamargine to 48 h showed similar results in terms of cells in the late apoptosis phase (1.2 and 2.9 folds, respectively) and overall killing effect (1.5 and 3.3 folds, respectively) (Fig. 7-B,D). It is worth to mention that both solasodine and solamargine induced significant necrosis which was indicative of non-specific killing effect (Fig. 7).

## Discussion

Developing countries are experiencing increasing rates of liver cancer. HCC is one of the three most commonly diagnosed cancers in Egypt that has a high mortality rate and limited therapeutic opportunities. Current mortality due to liver cirrhosis and cancer is over 40,000/year and is increasing annually^19^. Glycoalkaloids are interestingly biologically anticancer active compounds which can be found in several natural herbs such as *Solanum melongena*^18^. Herein and as a continuation of our previous work^9^, we explored the detailed antiproliferative/cytotoxic profile of three biologically active glycoalkaloids (solasonine, solasodine and solamargine) isolated from *Solanum melongena* against resistant and sensitive liver cancer cells (HepG2 and Huh7).

In the current study, we examined the HPLC profile of the total methanolic extract of *Solanum melongena* in terms of glycoalkaloids abundance. Solasodine, solasonine, and solamargine were observed, identified and quantified in the extract using ESI-MS technique. Yet, the underlying mechanisms of the observed *in vitro* cytotoxic activity of *Solanum melongena* total extract and the isolated glycoalkaloids against HepG2 cell lines was unclear^9^. Potential anticancer property of a compound might be attributed to its antiproliferative effects or direct cytotoxic activity^20^. We studied these two proposed modes of action for the isolated glycoalkaloids (solasodine, solasonine, and solamargine) using cell cycle analysis and annexin-V apoptosis assessment. In several cases, excessive/prolonged cell cycle arrest results in cell death and ultimately cytotoxicity^21–23^.

In the current work, all isolated glycoalkaloids induced obvious antiproliferative effect against Huh7 cells which could be attributed to the significant cell cycle arrest at S-phase. This cell cycle arrest was extended over 48 h and was most prominent after treatment with solamargine. Many compounds of natural origin exert their anticancer properties via S-phase arrest and cell cycle catastrophe^24^. Not only S-phase arrest, but also did solamargine induce cell cycle arrest at G_2_/M phase. This explains the decreased cell population in the supraG_2_ phase. Extended cell cycle arrest induced by solamargine and to a lesser extent by solasodine induced cell killing effect which could be observed by elevated cell population in the pre-G phase. Besides, the three glycoalkaloids influenced cell cycle progression of HepG2 cells similar to Huh7 cells; solasosonine, solasodine and solamargine induced significant cell cycle arrest at S-phase with compensatory cell accumulation in either G_0_/G_1_ and/or G_2_/M-phases. Yet, cell cycle arrest induced by solasonine and solamargine were relieved after extended exposure for 48 h. This could be explained by eliminated arrested cells by apoptosis cell death^25^. In addition, accumulation of cells in Pre-G phase, which is indicative of cell death, due to cell cycle arrest in HepG2 cells was much lower than Huh7 cells. HepG2 liver cancer cells were found before to be more resistant to anticancer treatment compared to Huh7 cells^26^; and other tumor cell lines as well^20^.

Programmed cell death induced by chemotherapy against cancer cells is preferred than non-pragrammed cell death^27^. Generally, the three glycoalkaloids under investigation increased pre-G cell cycle phase to different extends which is indicative of cell killing effect. Yet, we examined whether this killing effect is programmed or non-programmed in nature. Interestingly, all the three glycoalkaloids induced significant apoptosis in Huh7 cells; and minimal necrosis (non-programmed cell death) was induced only by solasodine. With respect to the more resisitant liver cancer cell line (HepG2), only solasodine and solamargine induced significant apoptosis and necrosis cell death. It was previously reported that glycoalkaloids (such as solasodine) showed better anticancer activities than their aglycone^28^. It was explained by the ability of rhamnose sugar moity to mediate binding to the cancer cells receptors^29^. Yet, the exact apoptosis mechanism and its underlying molecular pathways are strongly recommended for further investigation.

## Conclusion

Herein, the composition of the glycoalkaloid-rich extract of *Solanum melongena* was authenticated by HPLC and purified to be used as a chemotherapeutic agent. The current study confirms the antiproliferative anticancer activity of three naturally occurig glycoalkaloids (solasonine, solasodine and solamargine) isolated from the methaolic extract of the fruit peels of *Solanum melongena* against two liver cancer cell lines of different susceptibility. Particularly, solamargine was the most potent among the isolated glycoalkaloids followed by solasodine; and the least active anticancer glycoalkaloid was solasonine. The isolated glycoalkaloids induced antiproliferative effect which is attributed to inhibiting cell cycle progression in S-phase; this in turn induced progressive cell apoptosis and ultimately cell death. These compounds worth further investigation as potential therapeutic leads for anticancer drug development.

## Materials and Methods

### Extraction and isolation

The methanolic extract of the fruits peels (MEP) as well as the compounds; solasonine, solasodine and solamargine were prepared/or isolated by the authors as previously described^9^.

### Analysis of the MEP of *Solanum melongena* fruit peels (MEP) using RP-HPLC

#### Sample preparation

The MEP (20 mg) was dissolved in 1 ml of 40% methanol in H_2_O, using an ultrasonic, and loaded on an EXtrelut® prepacked column (Merck). Elution was carried out using 7 × 1 ml, 50% methanol in H_2_O, and the volume completed to 10 ml in a volumetric flask.

### HPLC method

Samples were analyzed using Waters 2695 HPLC system equipped with: a photodiode array detector (PDA) UV detector, Masslynx V4.1 SCN 714 software, and analytical BDS-Hypersil C18 column (250 × 4.6 mm i.d.; particle size 5 μm; Thermo). The analyses were carried out employing a gradient elution system. Solvent A was sodium phosphate buffer (pH 7.2; 0.01 M) and solvent B was acetonitrile. The gradient elution program was employed at a flow-rate of 1.0 mL/min, consisting of: 0–2 min (20% B, isocratic); 2–26 min (70% B, linear gradient); 26–30 min (85% B, isocratic, washing column); 30–31 min (20% B, linear gradient); 31–35 min (20% B, isocratic, column equilibration). A 10 μL of 5 mg/mL of MEP was injected. The following standards were injected to verify the identity of the eluted peaks: 1 μL of 5 mg/mL of solasodine standard, 5 μL of 4 mg/mL of solasonine standard, and 5 μL of 7 mg/mL of solamargine standard. Additionally, each standard was directly infused into MS to double confirm the identity of the standards. ESI-MS of the isolated compounds was detected from m/z 50 to 1000 using a MS QQQ mass spectrometer equipped with an electrospray ion source in positive ion mode. The following instrument settings were used; nebulizer gas, nitrogen, 40 psi; dry gas, nitrogen, 10 ml/min, 300 °C; capillary, −3000 V (+4000 V); end plate offset, −500 V; funnel 1 RF, 200 Vpp; funnel 2 RF, 200 Vpp.

### Cell culture

Human liver cancer cell lines (HepG_2_ and Huh-7) were obtained from the Vacsera (Giza, Egypt). Cells were maintained in DMEM culture medium, supplemented with 100 µg/ml streptomycin, 100 units/ml penicillin and 10% heat-inactivated fetal bovine serum. Cells were kept passaging in subconfluent state in humidified 5% CO_2_ (v/v) atmosphere at 37 °C as previously described^30^.

### Cytotoxicity assessment

The cytotoxicity of the isolated glycoalkaloids was tested against Huh-7 and HepG2 liver cancer cells by SRB assay as previously described^31^. Briefly, exponentially growing cells were trypsinized by 0.25% Trypsin-EDTA and seeded in 96-well plates at 1000–2000 cells/well. Cells were treated with serial concentrations of the isolated compounds for 72 h and subsequently fixed with TCA (10%) for 1 h at 4 °C. After several washings with water, cells were stained with 0.4% SRB solution for 10 minutes at room temperature in dark place and subsequently washed with 1% glacial acetic acid. After drying the plates overnight, Tris-HCl was used to dissolve the SRB stained cells and color intensity was measured at 540 nm with microplate reader (Spectramax® M3, Molecular devices, San Jose, CA, USA. Each concentration was replicated 6 times and the whole experiment was repeated three times; data represents mean ± SD of three replicates.

### Data analysis

The dose-response curves were analyzed as previously described^20^ using E_max_ model (Eq. 1).

1 $$ \% \mathrm{Cell}\,{\rm{viability}}=({\rm{100}}-{\rm{R}})\times ({\rm{1}}-\frac{{[{\rm{D}}]}^{{\rm{m}}}}{{{{\rm{K}}}_{{\rm{d}}}}^{{\rm{m}}}+{[{\rm{D}}]}^{{\rm{m}}}})+{\rm{R}}$$

Where [R] is the residual unaffected fraction (the resistance fraction), [D] is the drug concentration used, [K_d_] or IC_50_ is the drug concentration that produces a 50% reduction of the maximum inhibition rate and [m] is a Hill-type coefficient. Absolute IC_50_ is defined as the drug concentration required to reduce absorbance by 50% of control group (i.e., K_d_ = absolute IC_50_ when R = 0 and E_max_ = 100 − R).

### Cell cycle analysis

To assess the effect of isolated alkaloids (solasonine, solasodine and solamargine) on cell cycle distribution, cells were incubated with 5 µM of the test compounds for 24 h and 48 h. Cells were collected by trypsinization; washed twice with ice-cold PBS and re-suspended in 0.5 ml PBS. Two milliliters of 70% ice-cold ethanol was added gently while shaking. Cells were kept in ethanol solution at 4 °C for 1 hour for fixation. Upon analysis, cells were washed and re-suspended in 1 ml of PBS containing 50 μg/mL RNAase A and 10 μg/mL propidium iodide (PI). After 20 minutes incubation in dark place at room temperature, cells were analyzed for DNA contents by ACEA Novocyte™ flowcytometer (ACEA Biosciences Inc., San Diego, CA, USA) and analyzed for PI fluorescent signals using FL2 detector (λ_ex/em_ 535/617 nm). For each sample, 12,000 events were acquired. Percent of cells in each cell cycle phase was analyzed and calculated using ACEA NovoExpress™ software (ACEA Biosciences Inc., San Diego, CA, USA). Each treatment was repesated three times and data represents mean ± SD of three replicates.

### Apoptosis assessment using annexin V-FITC/PI staining coupled with flowcytometry

To assess the effect of isolated alkaloids on programmed cell death, apoptosis and necrosis cell populations were determined using Annexin/V-FITC apoptosis detection kit (Abcam Inc., Cambridge Science Park, Cambridge, UK). Briefly, cells were treated with 5 µM of solasonine, solasodine and solamargine for 24 h and 48 h. After treatment, cells were collected by trypsinization, washed twice with ice-cold PBS, and re-suspended in 0.5 mL of annexin/V-FITC/PI solution for 30 min in dark according to manufacturer protocol. After staining at room temperature, cells were injected through ACEA Novocyte™ flowcytometer (ACEA Biosciences Inc., San Diego, CA, USA) and analyzed for FITC and PI fluorescent signals using FL1 and FL2 signal detector, respectively (λ_ex/em_ 488/530 nm for FITC and λ_ex/em_ 535/617 nm for PI). For each sample, 12,000 events were acquired and positive FITC and/or PI cells were quantified by quadrant analysis and calculated using ACEA NovoExpress™ software (ACEA Biosciences Inc., San Diego, CA, USA). Each treatment was repesated three times and data represents mean ± SD of three replicates.

### Statistical analysis

Data are presented as mean ± SD. Analysis of variance (ANOVA) followed by Tukey’s post hoc test was used for testing the significance using SPSS for windows, version 17.0.0. p-value of 0.05 was taken as a cut off value for significance.
